# Translational formulation of nanoparticle therapeutics from laboratory discovery to clinical scale

**DOI:** 10.1186/s12967-019-1945-9

**Published:** 2019-06-14

**Authors:** Jie Feng, Chester E. Markwalter, Chang Tian, Madeleine Armstrong, Robert K. Prud’homme

**Affiliations:** 1 Department of Mechanical Science and Engineering, University of Illinois at Urbana-Champaign, Urbana, IL 61801 USA; 2 Department of Chemical and Biological Engineering, Princeton University, Princeton, NJ 08544 USA

**Keywords:** Translational medicine, Nanoparticles, Lumefantrine, Flash NanoPrecipitation, Bioavailability, Scale-up, Spray drying, Malaria

## Abstract

**Background:**

“Nanomedicine” is the application of purposely designed nano-scale materials for improved therapeutic and diagnostic outcomes, which cannot be otherwise achieved using conventional delivery approaches. While “translation” in drug development commonly encompasses the steps from discovery to human clinical trials, a different set of translational steps is required in nanomedicine. Although significant development effort has been focused on nanomedicine, the translation from laboratory formulations up to large scale production has been one of the major challenges to the success of such nano-therapeutics. In particular, scale-up significantly alters momentum and mass transfer rates, which leads to different regimes for the formation of nanomedicines. Therefore, unlike the conventional definition of translational medicine, a key component of “bench-to-bedside” translational research in nanomedicine is the scale-up of the synthesis and processing of the nano-formulation to achieve precise control of the nanoscale properties. This consistency requires reproducibility of size, polydispersity and drug efficacy.

**Methods:**

Here we demonstrate that Flash NanoPrecipitation (FNP) offers a scalable and continuous technique to scale up the production rate of nanoparticles from a laboratory scale to a pilot scale. FNP is a continuous, stabilizer-directed rapid precipitation process. Lumefantrine, an anti-malaria drug, was chosen as a representative drug that was processed into 200 nm nanoparticles with enhanced bioavailability and dissolution kinetics. Three scales of mixers, including a small-scale confined impinging jet mixer, a mid-scale multi-inlet vortex mixer (MIVM) and a large-scale multi-inlet vortex mixer, were utilized in the formulation. The production rate of nanoparticles was varied from a few milligrams in a laboratory batch mode to around 1 kg/day in a continuous large-scale mode, with the size and polydispersity similar at all scales.

**Results:**

Nanoparticles of 200 nm were made at all three scales of mixers by operating at equivalent Reynolds numbers (dynamic similarity) in each mixer. Powder X-ray diffraction and differential scanning calorimetry demonstrated that the drugs were encapsulated in an amorphous form across all production rates. Next, scalable and continuous spray drying was applied to obtain dried powders for long-term storage stability. For dissolution kinetics, spray dried samples produced by the large-scale MIVM showed 100% release in less than 2 h in both fasted and fed state intestinal fluids, similar to small-batch low-temperature lyophilization.

**Conclusions:**

These results validate the successful translation of a nanoparticle formulation from the discovery scale to the clinical scale. Coupling nanoparticle production using FNP processing with spray drying offers a continuous nanofabrication platform to scale up nanoparticle synthesis and processing into solid dosage forms.

**Electronic supplementary material:**

The online version of this article (10.1186/s12967-019-1945-9) contains supplementary material, which is available to authorized users.

## Background

Translation research refers to the “bench-to-bedside” enterprise of harnessing knowledge from basic sciences to produce new drugs, devices, and treatment options. For drug development, the end point is the production of a promising new treatment that can be used clinically or commercialized [1]. In the field of nanomedicine, one major bottleneck in the translation from bench to clinic is scale-up. Nanomedicine refers to the biomedical and pharmaceutical applications of nano-sized vehicles for the delivery of therapeutics, such as drugs, vaccines or genetic material [2]. Although the last few decades have witnessed the rapid progress in research on nanomedicine, scaling-up remains a significant barrier that delays the effective clinical adoption of nanoparticle (NP) formulation [3]. As Scott E. McNeil, the director of the Nanotechnology Characterization Laboratory at the U.S. National Cancer Institute has stated: “Another big hurdle in developing nanomedicines is scaling up the synthesis of the particles…developing a synthesis that yields particles with those precise properties on a consistent basis. That is still a difficult process.”

The major difficulty in NP scale-up is that scale-up dramatically alters the momentum and mass transfer rates that control NP assembly [4]. In one study of scaling up NP production using an emulsion method, Colombo et al. found that the increase in impeller speed and agitation time decreased the NP size [5], while another study by Galindo-Roderigue observed that the drug loading of NPs was reduced during scale-up from a laboratory batch volume of 60 mL to 1.5 L [6].

In this work, we demonstrate the scale-up of a nanoformulation process, called Flash NanoPrecipitation (FNP). FNP is a stabilizer-directed rapid precipitation process to produce NPs. In FNP, amphiphilic stabilizers and hydrophobic drugs are molecularly dissolved in an organic phase and mixed rapidly with an antisolvent stream to drive controlled precipitation with tunable particle size (~ 50–500 nm) and narrow size distribution [7, 8]. The reason that FNP scales well is that at all production scales the generation of supersaturation by turbulent micromixing is faster than the diffusion limited aggregation that controls NP assembly [9–11]. Variability in size and polydispersity is less than 10% over the entire composition range [9]. FNP has been used as a versatile and controllable platform to generate nanomedicines for parenteral administration as well as low-cost oral formulations. Previously we developed parenteral formulations with relatively expensive block-copolymer stabilizers [12–15]. Recently, we have been exploring the use of low-cost stabilizers in the formulation process, such as hydroxypropyl methylcellulose acetate succinate (HPMCAS), zein and lecithin, in order to enable affordable oral drugs for global health [16–19].

The successful scale up of NP formation overcomes only the first challenge in the path to a feasible oral dosage form. Equally important is to scale up the recovery process of the NPs into a dry, solid form without compromising the enhanced bioavailability [16]. Common techniques for solvent removal include lyophilization and spray drying. Lyophilization typically requires long processing time. While it is commonly used for high value parenteral drug formulation, it is problematic for large scale production of oral dosage forms. On the other hand, spray drying is a one-step, continuous, and scalable drying method [20]. Therefore, we focus on the utilization of spray drying to dry samples for large scale NP powder processing.

Lumefantrine (LMN), a hydrophobic anti-malaria drug with low oral bioavailability, was chosen as a model drug. In order to formulate affordable oral drugs for global health, we used a low-cost stabilizer, HPMCAS, which is a well-established pharmaceutical excipient [21]. Using appropriate mixers with various mixing geometry, we performed FNP using LMN and HPMCAS and examined the consistency of nanoparticles for different scales of production. Since FNP is a continuous process, larger batch sizes can be achieved with longer run time. However, to match downstream through-put requirements, larger mixers can also be employed. After the NP formulation, spray-drying was optimized to obtain dried powders, which were further characterized with powder X-ray diffraction (PXRD) and differential scanning calorimetry (DSC). Finally, the dissolution kinetics were tested in the simulated gastric and intestinal fluids for in vitro release from powders produced by the small scale and large scale mixers.

## Methods

### Materials

LMN was obtained as a gift from Medicines for Malaria Ventures. All solvents (HPLC grade) from Sigma-Aldrich (Milwaukee, WI) were used as received. AFFINISOL HPMCAS-126 (Additional file 1: Table S1) and METHOCEL HPMC E3 were gifts from Dow Chemical Company (Midland, MI). Fasted-state simulated intestinal fluid (FaSSIF), fed-state simulated intestinal fluid (FeSSIF-V2) and fasted-state simulated gastric fluid (FaSSGF) powders were purchased from Biorelevant.com (London, UK). Deionized (DI) water (18.2 MΩ cm) was prepared by a NANOpure Diamond UV ultrapure water system (Barnstead International, Dubuque, IA).

### Mixer design and fabrication

**Fig. 1:**
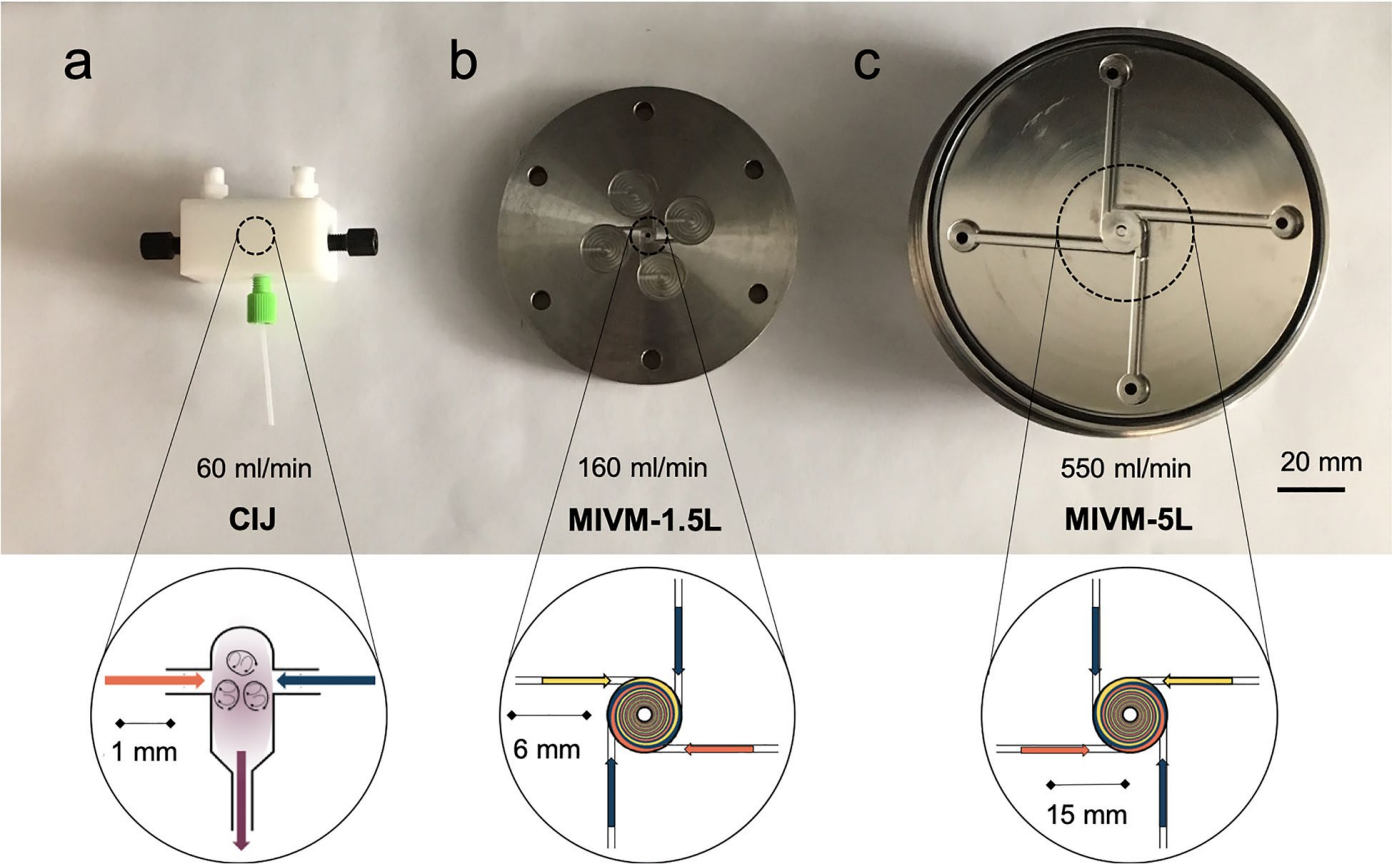
Images of the three mixers, including **a** confined impinging jet mixer (CIJ), **b** multi-inlet vortex mixer (MIVM)-1.5L and **c** MIVM-5L. Insets: zoom-in view of the mixing chambers of CIJ, MIVM-1.5L and MIVM-5L

Three kinds of mixers were used in the current study (Fig. 1). The confined impinging jet mixer (CIJ) can be used in a batch, hand-held mode with syringes to feed the device, which produces NP formulations with sub-milligram active pharmaceutical ingredient (API) requirements [22]. The CIJ can also be driven by syringe pumps to make samples with larger volume of 200–300 mL [10]. The geometry and operation of the device have been previously reported [7]. Furthermore, two multi-inlet vortex mixers (MIVM-1.5L and MIVM-5L) were also used to generate NP formulations. The MIVM’s four-inlet geometry allows higher supersaturation during mixing than the CIJ and bypasses the secondary quenching step [23]; therefore the MIVM mixer has advantages for continuous and large scale production. Both mixer geometries produce NPs of the same size and stability, as will be shown below. The MIVM naming convention is based upon the approximate outlet flowrate, in liters per minute, at a mixer Reynolds number of 10^5^. While the MIVM-1.5L (Fig. 1b) can be used to produce any batch size by scaling production time, nanoparticle processing often involves other unit operations such as tangential flow filtration or spray drying. The mixer size should be matched to the flows and time scales of the other unit operations [9, 24]. Therefore, to avoid operating under conditions where the mixing and assembly regime has changed, a larger MIVM with a higher flow rate may be used. We designed the MIVM-5L to operate at a volumetric flow rate of 5 L/min at Re = 10^5^ and used a modified form of the design reported by Markwalter and Prud’homme [24]. We adopted a strategy that constrained several parameters within boundaries reported by Liu et al. as well as Markwalter and Prud’homme [24, 25]. The MIVM-1.5L and MIVM-5L mixers are geometrically similar with the vortex chamber of the 5L design being 2.5 times larger than the 1.5L design presented by Liu et al. [26]. A two-disk design was used to simplify machining and mixer assembly. The mixer was fabricated from stainless steel 316L with an electropolished surface and 20 RA finish.

### Nanoparticle formulation and characterization

To optimize the NP formulations, nanoparticles were first created via a CIJ. An organic stream of tetrahydrofuran (THF) with molecularly dissolved LMN and HPMCAS, was rapidly mixed against a deionized (DI) water stream into the mixing chamber of a CIJ in a 1:1 volume ratio [22]. The concentration in the organic stream was 7.5 mg/mL for LMN and 3.75 mg/mL for HPMCAS. With the CIJ, fluid was pressed manually from syringes at the same rate (~ 1 mL in 1 s), causing the two streams to merge into a mixing stream. The flow rate through the mixer was approximately 120 mL/min. The resulting mixed stream was collected in a quenching DI water bath to lower the final THF concentration to 10 vol%. Lyophilization was used to dry the CIJ samples.

In the MIVM, one organic stream containing 7.5 mg/mL LMN and 3.75 mg/mL HPMCAS-126 was mixed against three other water streams, with a volumetric flow rate of 1:9 (organic:water in total). The final organic solvent concentration as 10 vol%. Process development was carried out in the MIVM-1.5L using syringe pumps, which is convenient for samples from 20 to 300 mL. We then implemented Coriolis flow controllers (M14, mini CORI-FLOW, Bronkhorst, NL) to demonstrate a continuous process. The MIVM-5L was only operated with the flow controllers. The total flow rate was 160 and 550 mL/min for MIVM-1.5L and MIVM-5L, respectively. Based on the nanoparticle concentration, the mass production rate of MIVM-5L is 1 kg/day. Higher flow rates can further increase the mass production rates [24]. The MIVM-5L is designed to produce LMN NPs at 8 kg/day with Reynolds number of 10^5^. Spray drying was used to dry the MIVM samples.

Using a Zetasizer Nano-ZS (Malvern Instruments, Southboro, MA), NP diameter and polydispersity index (PDI) were determined, in triplicate, by dynamic light scattering (DLS) at 25 °C with a detection angle of 173°. DLS data were processed with Malvern’s software using a cumulant model for distribution analysis. The cumulant analysis is defined in International Organization for Standardization (ISO) standard document 13321. The calculations of PDI are defined in the ISO standard document 13321:1996 E.

### Transmission electron microscopy (TEM) imaging

Nanoparticle suspensions produced in either a CIJ or MIVM-1.5L were dropcast (~ 5 μL) onto a copper TEM grid (300 mesh carbon film, Electron Microscopy Sciences). Vapor-phase ruthenium staining was carried out by generating ruthenium tetroxide from ruthenium dioxide using sodium meta-periodate. The grids were placed in a sealed container with aqueous ruthenium solution until a cellulose sample indicated sufficient staining. Micrographs were obtained using a Philips CM-200 FEG-TEM at an accelerating voltage of 200 kV.

### Nanoparticle lyophilization

Lyophilization was carried out using a benchtop VirTis Advantage (Gardiner, NY) with appropriate cryoprotectants (HPMC E3). In our previous study with clofazimine [16, 17], HPMC E3, a water-soluble HPMC polymer, was used for HPMCAS NPs. The HPMC E3 serves as a cryoprotectant and prevents aggregation between the HPMCAS NPs during freezing and drying. 1 mL NP suspension were mixed with 0.1 mL cryoprotectant solutions to reach a 1:1 mass ratio of NP:cryoprotectant. The mixtures were then flash frozen by fast immersion in a dry ice/acetone cooling bath (− 78 °C) for 1 min with mild agitation. The frozen samples were then immediately transferred to the lyophilizer with shelf temperature at − 20 °C under vacuum (< 1 × 10^−3^ bar). After 2 days, dried powders were removed, sealed, and stored at − 20 °C. Lyophilization was only used for NP suspension generated by CIJ as the baseline for dissolution test.

### Spray drying

A mini spray-drier B-290 (BÜCHI Corporation, New Castle, DE), equipped with a two-fluid nozzle, was used for drying the NP suspension in an open mode. After FNP, the NP suspension was mixed with the excipient, HPMC E3, at a mass ratio of 1:1. The suspension was then fed by a peristaltic pump into the spray-drier. The spray nozzle consisted of a tip and a cap with diameter of 0.7 and 1.5 mm, respectively, and the drier was equipped with a high-performance cyclone provided by BÜCHI. Compressed nitrogen at 480 kPa was used to atomize the liquid phase into droplets, and the flow rate was controlled by a rotameter. The inlet temperature, outlet temperature, drying gas flow rate, liquid feed rate and the gas flow rate of the aspirator were shown in Table 1. Spray dried powders were collected in scintillation vials, sealed, and stored at a vacuum desiccator and room temperature (20 °C) before use.

**Table 1:** Spray drying conditions for NP formulations, including the inlet temperature, outlet temperature, spray gas flow rate, sample feed rate, aspiration flow rate

T_inlet_ (°C)	T_outlet_ (°C)	Sample feed rate (mL/min)	Drying gas flow rate (L/h at standard temperature and pressure)	Aspiration flow rate (m^3^/h at standard temperature and pressure)
100	46	5	350	35

### Powder X-ray diffraction

PXRD was performed using a Bruker D8 Advance Twin diffractometer equipped with Ag Kα radiation (λ = 0.56 Å) and LYNXEYE-XE detector. In each test, approximately 10 mg of powder was loaded into a polyimide capillary with an inner dimeter of 1 mm. Then the tube was mounted on a capillary stage, which rotated at a speed of 60 rpm during operation. Signals were collected between values of 3°–20° (2*θ*, corresponding to a Cu Kα 2*θ* value of ~ 8°–58°) with a step size of 0.025° (0.070° for Cu Kα radiation) and a count rate of 5 s/step. All PXRD results are presented with 2*θ* value corresponding to a Cu Kα radiation.

### Differential scanning calorimetry (DSC)

DSC experiments were performed with a TA Instrument Q200 (New Castle, DE) with hermetically sealed aluminum pans. Dried samples (5–10 mg) were equilibrated at 20 °C under dry N_2_ atmosphere (50 mL/min), and then heated from 20 to 200 °C at a heating rate of 5 °C/min. The scan was analyzed by TA Instruments Universal Analysis 2000 software.

### Dissolution test

FaSSGF, FaSSIF and FeSSIF buffers were prepared following the manufacturer’s instructions. Triplicate experiments were performed for each sample, and free LMN powder was used as a control. For release under gastric conditions, dried powders were first resuspended in water and then diluted with pre-warmed FaSSGF (37 °C) to achieve a drug concentration of 50 μg/mL. The suspensions were then incubated at 37 °C (NesLab RTE-111 bath circulator, Thermo Fisher Scientific, Waltham, MA) for 30 min without agitation to mimic physiological gastric conditions and transit time in the stomach [27]. Since Brownian motion kept the small particles well dispersed, the effect of gastric mixing was not considered. Aliquots were taken at 5, 10, 20, and 30 min, which was centrifuged at 21,000*g* for 10 min to pellet NPs. For release under intestinal conditions, the solutions after the FaSSGF protocol were diluted 10× with 1.1× FaSSIF (pH = 6.5) or FeSSIF (pH = 5.8) with a final LMN concentration lower than its solubility limit in both buffers. Aliquots were taken at 30, 60, 120, 240, and 360 min, and were centrifuged at 21,000*g* for 10 min. Centrifugation provides complete separation of the nanoparticles from the supernatant, as confirmed by the lack of DLS signal in the supernatant after centrifugation. All supernatants were then removed, frozen, and lyophilized for later tests, and the sampling time points were defined as the incubation time from the assay start to the sampling.

### High performance liquid chromatography

High performance liquid chromatography (HPLC) was used to analyze the supernatants from the dissolution tests with a Gemini C18 column (particle size 5 μm, pore size 110 Å). The dried powder from the supernatants was resuspended in a mixture of acetonitrile (ACN) and THF (90/10, v/v), and then further sonicated to dissolve LMN. To pellet the insoluble bile salts from the buffers, each aliquot was centrifuged at 21,000*g* for 3 min. The supernatant was then filtered through a GE Healthcare Life Sciences Whatman™ 0.1 µm syringe filter. An isocratic mobile phase of ACN:water (60/40, v/v, both with 0.05 vol% trifluoroacetic acid) at 45 °C was applied to detect LMN with a flow rate of 1 mL/min. The LMN peak at 347 nm eluted at 6.8 min. The standard curve linearity was verified from 25 to 0.5 μg/mL with an *r*^2^ value of at least 0.999 (Additional file 1: Figure S1).

## Results

### Nanoparticle formulations by CIJ and MIVM

**Fig. 2:**
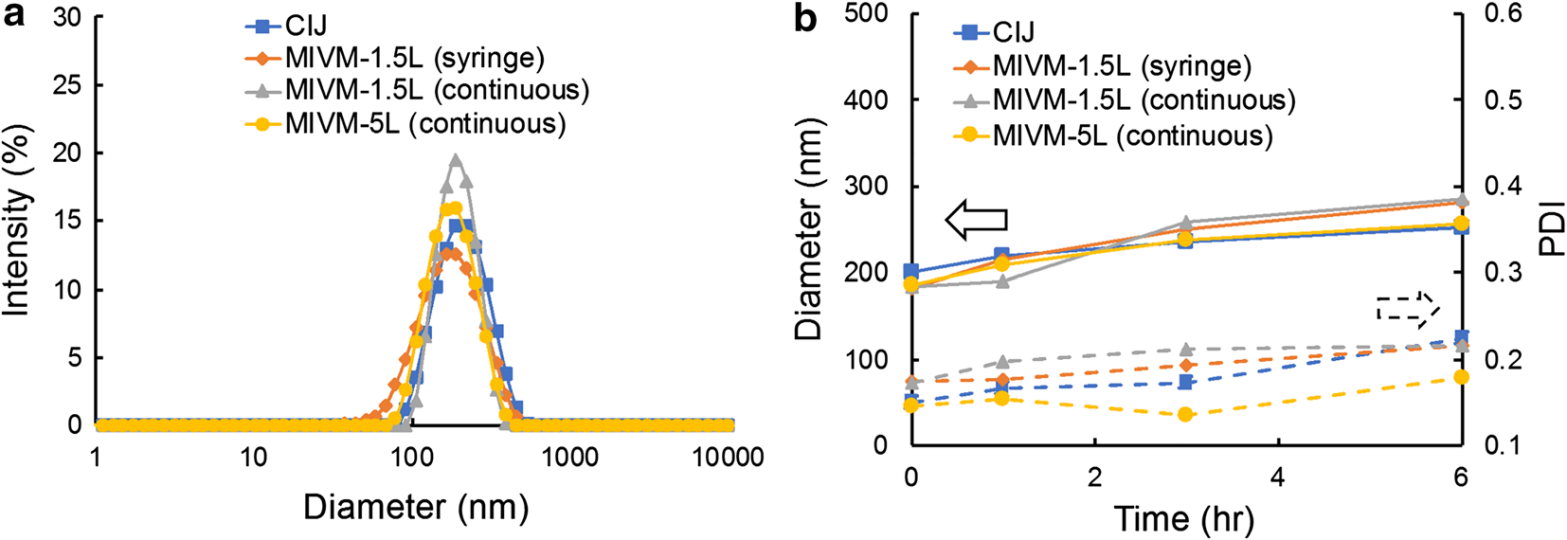
**a** NP diameter after FNP and **b** size stability of NPs formed by different mixers, including CIJ, MIVM-1.5L with syringe pumps or continuous flow controllers and MIVM-5L with continuous flow controllers. *CIJ* confined impinging jet mixer, *MIVM* multi-inlet vortex mixer, *PDI* polydispersity

**Fig. 3:**
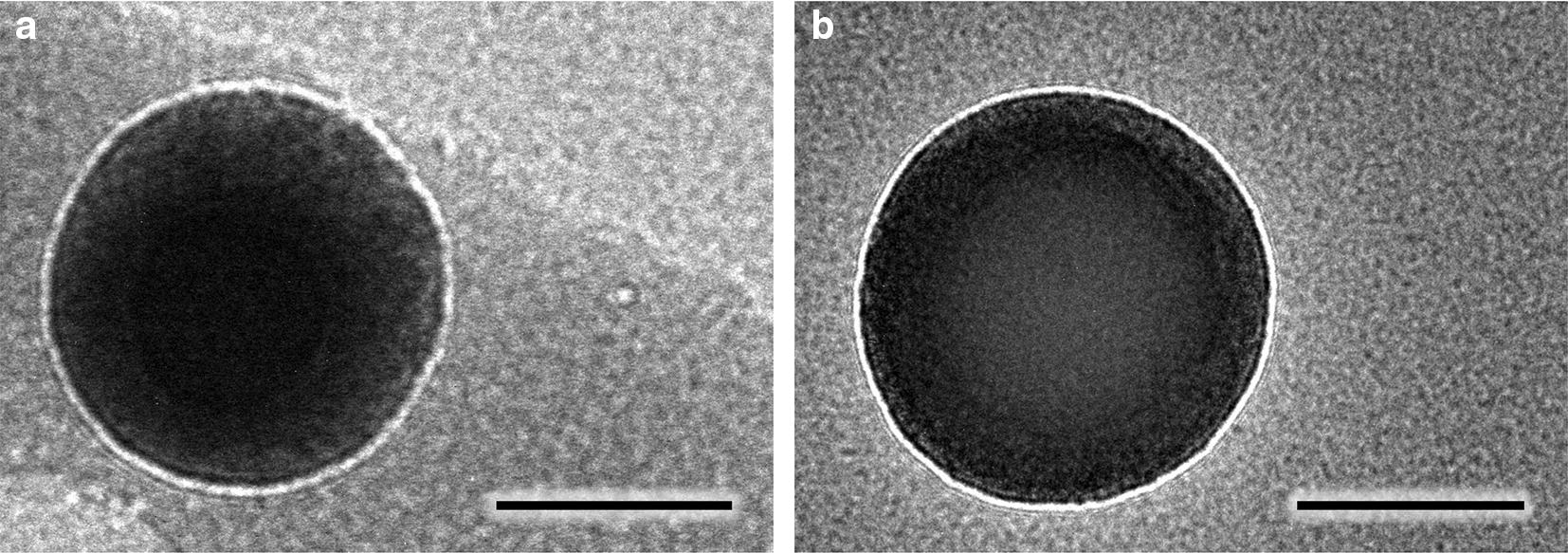
TEM images of **a** nanoparticles produced by CIJ and **b** nanoparticles produced by the MIVM-1.5L (continuous). Scale bars are 100 nm. Nanoparticles were stained with ruthenium. Images are representative of the grid after inspection

With the same formulation, we performed FNP by utilizing the CIJ, MIVM-1.5L and MIVM-5L mixers. For the MIVM-1.5L, we used both syringe pumps and Coriolis flow controllers to demonstrate the transition from a batch to continuous processing. The flow rate was increased with the chamber size to keep similar Reynolds numbers across different mixers, so that the time scale of turbulent micromixing was similar. Scale-up studies on the CIJ mixer have shown that geometric scaling results in identical mixing times [10]. As demonstrated in Fig. 2a, the NPs generated through different approaches shows a consistent size distribution of around 200 nm, with less than 8% difference in NP sizes for four different mixing processes. The NPs show some slow increase in size resulting from Ostwald ripening over 6 h (Fig. 2b). In addition, TEM images indicate spherical particles in line with the size distributions measured by DLS. Particles made by CIJ or MIVM at different scales were indistinguishable, as shown by representative images in Fig. 3. We designed the NP formulation followed by spray drying to occur over less than 3 h. The size stability allows sufficient time for processing into dry powders.

### Spray drying of lumefantrine nanoparticles

**Fig. 4:**
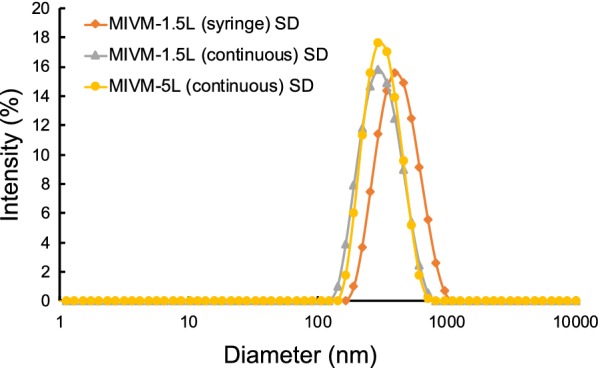
Redispersion by water of the spray-dried samples from different mixers. *CIJ* confined impinging jet mixer, *MIVM* multi-inlet vortex mixer

Table 1 summarizes the optimized spray drying parameters, including the inlet gas temperature, outlet gas temperature, sample feed rate, drying gas flow rate and aspiration flow rate. Since LMN has a low melting point of 128–131 °C [28], we selected an inlet gas temperature of 100 °C. All spray dried particles had low levels of residual moisture content below 2 wt%, which indicates that spray drying removed the solvents effectively. Furthermore, as shown in Fig. 4, the spray dried powders are easily redispersed in water to NPs with a size distribution between 300 and 400 nm, indicating no irreversible particle aggregation during spray drying. The maintenance of nanoscale size is important since the high surface-to-volume ratio of the NPs contributes to fast dissolution [29].

### PXRD and DSC

**Fig. 5:**
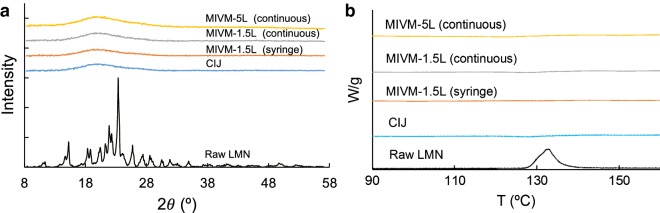
**a** Powder X-ray diffraction and **b** differential scanning calorimetry for dried samples from different mixers. *CIJ* confined impinging jet mixer, *MIVM* multi-inlet vortex mixer, *LMN* lumefantrine

PXRD and DSC were used to characterize the physical state of a drug in a polymeric matrix. A CIJ sample dried by lyophilization was the baseline for comparison in the following discussions. In PXRD (Fig. 5a), the diffractogram of the raw LMN powder consists of sharp Bragg peaks, corresponding to the bulk crystalline nature of the drug. However, all dried NP powders showed no indication of crystallinity, Additionally, the encapsulated LMN is in an amorphous form as confirmed by 2D solid state nuclear magnetic resonance measurement [19]. The broad peak at 2*θ* = 20° is from the amorphous cellulosic polymers. In the DSC thermogram (Fig. 5b), the raw LMN powder is characterized by a single, sharp peak at 132 °C. Complete disappearance of the melting endotherm in the DSC scan of all the dried NP samples also shows that a substantially amorphous state of LMN was produced in the FNP process. Comparing the CIJ and MIVM samples, no difference of PXRD and DSC signals can be identified in Fig. 5. Therefore, in the scale-up process, the amorphous state of the encapsulated LMN was preserved.

### Dissolution tests

Pharmaceutical solid dosage forms must undergo dissolution in the intestinal fluids of the gastrointestinal tract before the drugs can be absorbed. LMN is practically insoluble in water (log *P *= 9.19) [30], but has high permeability. Consequently, the key determinant in the bioavailability of LMN is the dissolution rate [31]. To demonstrate the consistency of the NPs produced by mixers at different scales, we performed experiments to test the in vitro LMN dissolution kinetics for dried powders produced using the different mixers. The solubility of crystalline LMN in FaSSGF, FaSSIF, and FeSSIF was determined to be 0.51, 4.8, and 14 μg/mL, respectively.

**Fig. 6:**
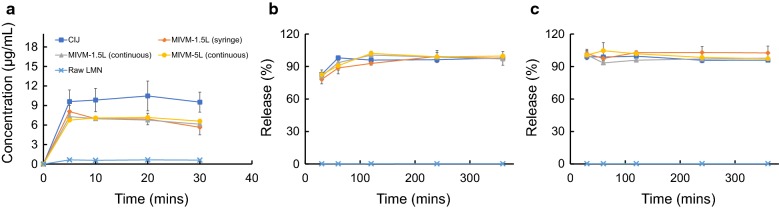
Dissolution kinetics in **a** fasted state simulated gastric fluid, **b** fasted state simulated intestine fluid and **c** fed state simulated intestine fluid for dried samples from different mixers. *CIJ* confined impinging jet mixer, *MIVM* multi-inlet vortex mixer, *LMN* lumefantrine

To study the dissolution in FaSSGF, NP samples were dispersed in water and then diluted into FaSSGF with an initial concentration of 100× the equilibrium solubility of crystalline LMN. LMN powder was included as the control sample. Through a 30-min incubation at 37 °C, the concentration evolution of LMN dissolved in the FaSSGF from various samples is shown in Fig. 6a. As expected, the crystalline LMN only reached the solubility limit of 0.51 μg/mL. All NPs reach their maximum drug concentrations after 5-min incubation, and these maximum concentrations are more than 12× the equilibrium solubility of crystalline LMN. The increase in solubility of NPs is attributed to the amorphous state of the drug [32]. All spray dried samples from MIVM-1.5L and MIVM-5L achieved similar supersaturation levels, which were only slightly lower than that of the lyophilized CIJ sample. The drop in supersaturation after 20 min was caused by the recrystallization of the dissolved LMN.

Next, after the 30-min initial exposure at 37 °C and pH = 1.6 to simulate stomach conditions, the NP/gastric fluid solution was further diluted into FaSSIF or FeSSIF to simulate the fasted or fed state conditions in the gastrointestinal tract, respectively. The dissolution kinetics of the LMN formulations at different time points are shown in Fig. 6 (b) FaSSIF and (c) FeSSIF. Here, the percentage of release is defined as the mass ratio between the dissolved drug and the total drug in the assay. The extremely low bioavailability of LMN is indicated by the slow release of the crystalline LMN (less than 1% in 6 h). In contrast, all NP samples exhibited a much faster release in both simulated intestinal fluids, showing almost 100% release after just 1 h in both FaSSIF and FeSSIF. No release difference was observed between the lyophilized CIJ and spray dried MIVM samples in intestinal fluid. The release profiles were similar across processing scales: from the small scale lyophilized CIJ NPs to the large scale, continuous spray dried MIVM NPs.

## Discussions

To demonstrate the feasibility of FNP as a scale-up technique for translational nanomedicine, we used a model drug, LMN, with a low-cost stabilizer, HPMCAS to formulate NPs. Three mixers, designed for different production rates (from laboratory scale of mg/day to pilot-plant scale of kg/day), were used in NP production. As demonstrated in Fig. 2, the produced NPs show the same sizes and polydispersities, with stability up to 6 h.

Furthermore, a continuous and scalable drying process, spray drying, was successfully used to produce solid dosage forms of NP powders. The hot and humid climates in tropical and equatorial regions could induce recrystallization of the encapsulated drug, in particular when solvent is present [33]. The utilization of spray drying to produce NP powders opens a path to provide improved long-term storage stability compared with NP suspensions, which is critical for translational research of therapeutic NPs for global health. After spray drying, the redispersity with water and in vitro dissolution kinetics were similar for powders produced at a small scale by lyophilization and at a large scale by spray drying. Characterization with PXRD and DSC indicates that the encapsulated drug maintained a low crystallinity level across all production scales and drying processes. Such consistency between NP samples using a bench-scale device and a clinical-scale mixer highlights the potential of the FNP processing to solve the scale-up issue associated with the translational research of nanomedicines.

## Conclusions

The highly hydrophobic LMN requires nanoparticle formulation in an amorphous state to produce high supersaturations and bioavailability. We successfully made LMN-loaded NPs of 200 nm using FNP at all three scales of mixers, and solidified the NPs into dried powders by spay drying. The spray dried samples produced by the large-scale MIVM showed 100% release in less than 2 h in both fasted and fed state intestinal fluids. The release kinetics were similar whether the samples were made by the large scale MIVM followed by spray drying, or by the laboratory scale, hand-held CIJ mixing at the mL scale, followed by low-temperature lyophilization. The robustness of the FNP process suggests a continuous, integrated platform for nanomedicine, in such a way that NPs are produced continuously via FNP and fed in-line directly to a spray drying unit. In this configuration, production rates between unit operations must be matched. Scaling on the dimensionless Reynolds number has been demonstrated for the mixers [10, 24] and the large-scale spray drying is currently practical. Straightforward scale-up of the synthesis and processing of therapeutic nanoparticles into solid dosage forms may provide an efficient solution to enable the translation of a discovery-level nano-formulation into clinically-relevant dosage forms.

## Additional file

**Additional file 1: Table S1.** Specification of AFFINISOL™ HPMCAS. **Figure S1.** Calibration curve for lumefantrine dissolved in the mobile phase of HPLC at 347 nm.

## Data Availability

Not applicable.

## Abbreviations

FNP: Flash NanoPrecipitation
NP: nanoparticle
HPMCAS: hydroxypropyl methylcellulose acetate succinate
LMN: lumefantrine
PXRD: powdered x-ray diffraction
DSC: differential scanning calorimetry
FaSSGF: fasted-state simulated gastric fluid
FaSSIF: fasted-state simulated intestinal fluid
FeSSIF: fed-state simulated intestinal fluid
DI: deionized
CIJ: confined impinging jets
MIVM: multi-inlet vortex mixer
API: active pharmaceutical ingredient
THF: tetrahydrofuran
ISO: International Organization for Standardization
DLS: dynamic light scattering
PDI: polydispersity
TEM: transmission electron microscopy
HPLC: high performance liquid chromatography
