# Supplementary material for: Translational formulation of nanoparticle therapeutics from laboratory discovery to clinical scale
Source: J Transl Med. 2019 Jun 14;17:200. doi: 10.1186/s12967-019-1945-9 (PMC6570894; doi:10.1186/s12967-019-1945-9)
Supplement: Supplementary file 1 — Additional file 1: Table S1. Specification of AFFINISOL™ HPMCAS. Figure S1. Calibration curve for lumefantrine dissolved in the mobile phase of HPLC at 347 nm. [file 12967_2019_1945_MOESM1_ESM.docx]

**Additional File**

**Translational Formulation of Nanoparticle Therapeutics from Laboratory Discovery to Clinical Scale**

Jie Feng^1,2^, Chester E. Markwalter^2^, Chang Tian^2^, Madeleine Armstrong^2^,

Robert K. Prud’homme^2,*^

*1. Department of Mechanical Science and Engineering, University of Illinois at Urbana-Champaign, Urbana, Illinois 61801, United States*

*2. Department of Chemical and Biological Engineering, Princeton University, Princeton, New Jersey 08544, United States*

Author emails, respectively: [jiefeng@illinois.edu](mailto:jiefeng@illinois.edu), [cem3@princeton.edu](mailto:cem3@princeton.edu), [ctian@princeton.edu](mailto:ctian@princeton.edu), [marmsljca@gmail.com](mailto:marmsljca@gmail.com), [prudhomm@princeton.edu](mailto:prudhomm@princeton.edu)

**Information of AFFINSOL^TM^ HPMCAS**

Table S1. Specification of AFFINISOL^TM^ HPMCAS (<https://www.dow.com/en-us/pharma/products/affinisol)>

|  | HPMCAS 126 |
| --- | --- |
| Hydroxypropyl | 6.0-10.0% |
| Methoxyl | 22.0-26.0% |
| Viscosity^*^ | 2.4-3.6 cP |
| Residue on Ignition | <0.20% |
| Loss on Drying | <5.0% |
| Free Acids | <1.0% |
| Acetate Substitution | 10.0-14.0% |
| Succinate Substitution | 4.0-8.0% |
| Acetic Acid | 0.5% |

**Lumefantrine calibration curves in the mobile phase of HPLC at 347 nm**

The mobile phase of HPLC is ACN:water (60/40, v/v, both with 0.05 vol% trifluoroacetic acid)


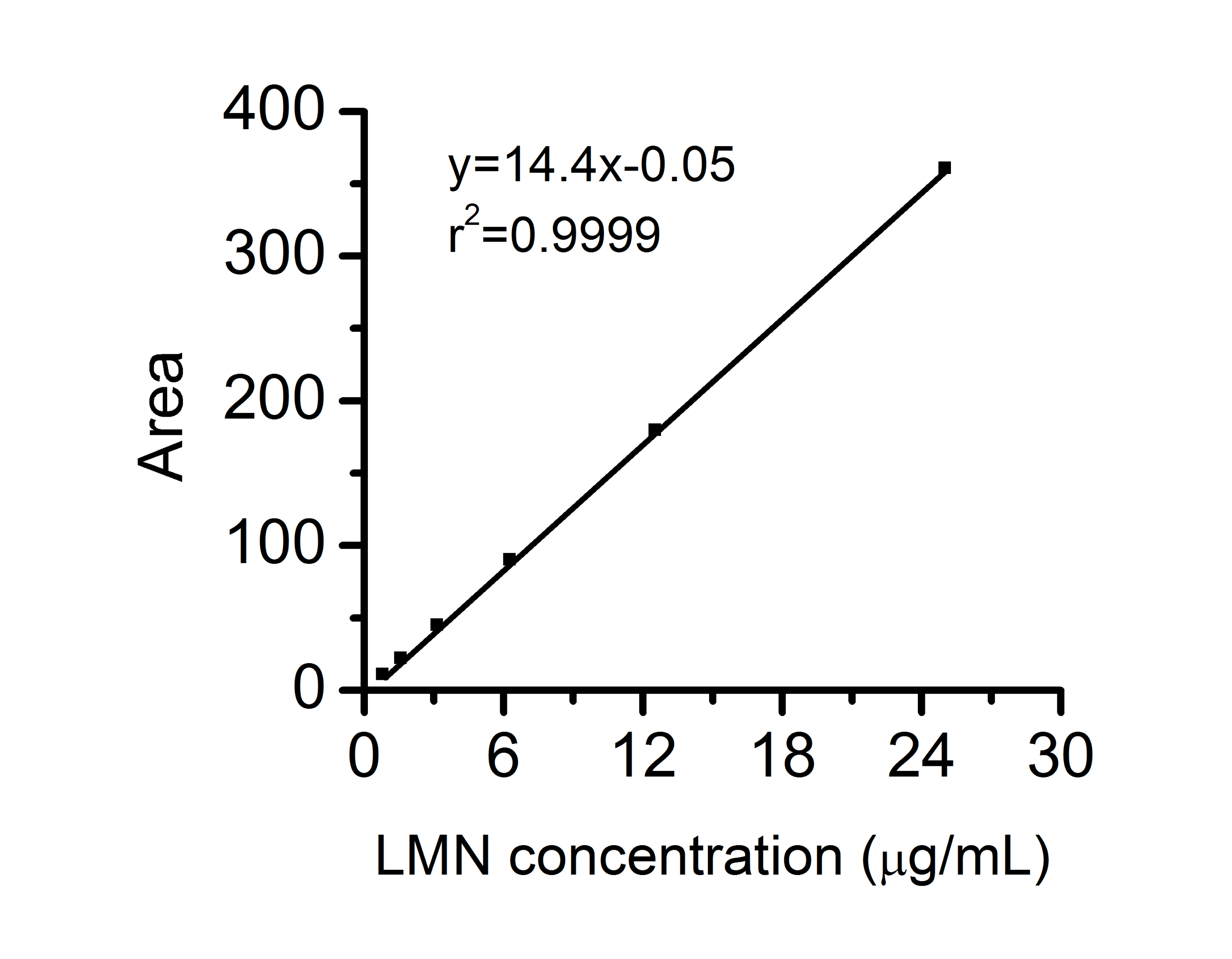


Figure S1. Calibration curve for lumefantrine dissolved in the mobile phase of HPLC at 347 nm
